# Real-world treatment patterns for atopic dermatitis in South Korea

**DOI:** 10.1038/s41598-022-17222-y

**Published:** 2022-08-10

**Authors:** Ji Hyun Lee, Ahhyung Choi, Yunha Noh, In-Sun Oh, Ja-Young Jeon, Hyun-Jeong Yoo, Ju-Young Shin, Sang Wook Son

**Affiliations:** 1 Department of Dermatology, Seoul St. Mary’s Hospital, College of Medicine, The Catholic University of Korea, Seoul, South Korea; 2 School of Pharmacy, Sungkyunkwan University, 2066 Seobu-ro, Jangan-gu, Suwon, Gyeonggi-do 16419 South Korea; 3 Department of Biohealth Regulatory Science, Sungkyunkwan University, Suwon, South Korea; 4 Pfizer Pharmaceuticals Korea Ltd., Seoul, South Korea; 5 Department of Clinical Research Design and Evaluation, Samsung Advanced Institute for Health Sciences and Technology, Sungkyunkwan University, Seoul, South Korea; 6 Department of Dermatology, Korea University College of Medicine, 46 Gaeunsa 2-gil, Seongbuk-gu, Seoul, 02842 South Korea

**Keywords:** Atopic dermatitis, Epidemiology

## Abstract

The phenotypes of atopic dermatitis (AD) are diverse, and ethnic differences have been suggested. To date, few studies have explored large-scale national data on the treatment patterns of AD in Asians. Therefore, we aimed to examine real-world treatment patterns for AD, including the probability of discontinuation of AD treatment and restart after discontinuation. A retrospective observational study was conducted using the nationwide healthcare database in South Korea between January 1, 2016 to July 31, 2020. We identified 944,559 pediatric patients and 1,066,453 adults with AD. Topical corticosteroids and antihistamines were the most commonly prescribed medications in all age groups. The frequency of topical corticosteroid prescription decreased as the age increased. Although immunosuppressive drugs were not widely used in both children and adults, cyclosporine was the most frequently prescribed immunosuppressant, particularly among those aged 12 years or more (1–2%). Pediatric patients were more likely to discontinue treatment than adult patients. Treatment restart for moderate-to-severe AD was earlier than that for overall AD. In conclusion, significant differences were observed in the treatment patterns of AD between pediatric and adult patients. These findings will improve our understanding of the latest treatment patterns for AD, which may contribute to decision-making in clinical practice.

## Introduction

Atopic dermatitis (AD), also known as atopic eczema, is a common chronic inflammatory skin disorder that affects 11–20% of children and 5–8% of adults. While the prevalence of AD has decreased or plateaued in some countries, its global prevalence is generally increasing^1,2^. The onset of AD is most common during the first five years of life; however, it can begin at any age and may persist for a long period, as the disease has a relapsing–remitting course with repeated acute flareups^3^. Indeed, the pathogenesis of AD is complex, involving genetic predisposition, immune dysfunction, and environmental factors, which poses therapeutic challenges to clinicians^4^.

To date, there is no definite cure for AD; thus, the treatment aims to manage the symptoms and reduce inflammation to improve the quality of life of patients. Current treatment guidelines for AD largely depend on its severity and the age of patients^5^. Topical corticosteroids (CSs) are recommended as the first-line treatment for flares and for controlling mild AD in both pediatric and adult patients. Systemic therapies, including cyclosporine and dupilumab, are approved for use in recalcitrant chronic AD. Meanwhile, off-label treatments have been frequently used for refractory AD, which is not well controlled by conventional treatments^6^. Accordingly, understanding the current prescription patterns for AD is important for addressing the gap between clinical practice and relevant guidelines. At present, there are limited data on real-world treatment patterns for AD, particularly in Asian populations^7^. The phenotypes of AD are diverse, and differences according to ethnicity have also been suggested^8^.

Herein, we aimed to describe real-world treatment patterns for AD, stratified by age group, using South Korea’s nationwide healthcare database. In addition, we evaluated the time to discontinuation of AD treatment and time to treatment restart after discontinuation.

## Materials and methods

### Study design and data source

This retrospective observational study was conducted using data collected from the Health Insurance Review and Assessment Service (HIRA) database in South Korea from January 1, 2016 to July 31, 2020. This database covers approximately 98% of the South Korean population and contains comprehensive information on healthcare services. It includes an individual’s anonymized identifier with demographic characteristics (i.e., age, sex, and health insurance type), inpatient and outpatient diagnoses based on the International Classification of Diseases 10th Revision (ICD-10), and information on drug prescriptions (i.e., prescription date, dosage, duration, and route of administration). This study was approved by the institutional review board of Sungkyunkwan University, South Korea (No. 2020-09-009). Because this study used anonymized administrative data, the requirement for informed consent was waived by the institutional review board of Sungkyunkwan University. All research was conducted in accordance with guidelines and regulations of the institutional and national research committee and with the 1964 Helsinki declaration.

### Study population

We identified all patients who were newly diagnosed with AD and subsequently received at least one prescription for AD treatment between January 1, 2017 and July 31, 2020. The date of the first prescription for AD treatment served as the index date. We excluded patients diagnosed with AD in 2016 to identify only incident patients diagnosed with AD within our study period. In addition, we excluded patients with diagnoses of immune-mediated inflammatory diseases during the 1-year period preceding the index date to avoid the possibility of including patients who were treated for immune-mediated diseases other than AD (Table S1). Lastly, patients who could not be followed up for at least 1 year from the index date were excluded.

We then constructed two cohorts based on age at the index date. Patients aged < 18 years on the index date were classified as the pediatric cohort and those aged ≥ 18 years at the index date were classified as the adult cohort.

### Treatment patterns

We defined the following medications to observe the treatment patterns of AD: topical CSs, topical calcineurin inhibitors (CIs), antihistamines, systemic CSs, immunosuppressants (cyclosporine, azathioprine, methotrexate, mycophenolate mofetil, and interferon-γ), intravenous immunoglobulin (IVIG), alitretinoin, montelukast, dupilumab, and phototherapy. Based on the World Health Organization classification of topical CSs, we categorized the medications into three levels: low (class 6–7), medium (class 3–5), and high (class 1–2)^9^. In South Korea, dupilumab was eligible for reimbursement for the indication of AD, as of January 1, 2020.

The prevalence of medication use at the index date (treatment initiation) and during the entire observation were examined. To exclude the possibility of potential use for related conditions other than AD, we restricted medications to prescriptions for primary or secondary AD diagnosis. In addition, as an exploratory analysis, we identified the factors associated with oral CSs use and treatment discontinuation.

### Discontinuation and restart of AD treatment

Furthermore, we assessed the time to treatment discontinuation and the time to restart after discontinuation in the pediatric and adult cohorts, respectively. Discontinuation of AD treatment was defined as the absence of a prescription for AD treatment for ≥ 6 months. Follow-up began on the index date and ended on the date of discontinuation or at the end of the study period. Restart of AD treatment was defined as the prescription of AD medication after the discontinuation of AD treatment (≥ 6 months gap).

Additionally, to observe treatment patterns focused on systemic treatments for moderate-to-severe AD, the discontinuation and restart of moderate-to-severe AD treatment were evaluated separately. Moderate-to-severe AD treatment was defined as treatment with at least one immunosuppressant, IVIG, alitretinoin, dupilumab, or phototherapy. Patients were followed up from their date of first moderate-to-severe AD prescription until the date of discontinuation or the end of the study period.

### Patient characteristics

Demographic characteristics, such as age, sex, insurance type, and region of residence, were assessed on the index date. Different comorbid conditions were measured within a 1-year period before the index date for pediatric and adult cohorts, as common comorbidities may differ between children and adults. Proxies of overall health status, including the duration of hospitalization, number of hospital visits, and Charlson comorbidity index score, were also evaluated for the period during the year before the index date^10^.

### Statistical analysis

Baseline characteristics are presented as frequency (proportion) for categorical variables and as mean (standard deviation, SD) or median (interquartile range, IQR) for continuous variables. To estimate the prevalence of AD medication at treatment initiation and during the entire observation period, the results were stratified by age group: 0–1, 2–5, 6–11 and 12–17 for the pediatric cohort and 18–39, 40–59 and ≥ 60 years for the adult cohort. The prevalence was calculated as the number of patients that were prescribed treatment for each medication class, where the denominator represented the total number of patients in each age group. The prevalence of AD medication use between the pediatrics and adults were compared using χ2 test or Fisher’s exact test for categorical variables and Student t-test for continuous variables. *p*-values of < 0.05 were defined as statistically significant.

To identify the factors associated with oral CSs use and treatment discontinuation, multivariable logistic regression was used to estimate the odds ratios (ORs) and 95% confidence intervals (CIs).

Kaplan–Meier survival curves were used to quantify the time to discontinuation and restart after the discontinuation of AD treatment. We compared the survival curves of the pediatric and adult cohorts using the log-rank test. All the analyses were performed using SAS, version 9.4 (SAS Institute).

## Results

### Patient characteristics

Among 4,872,859 patients diagnosed with AD between January 1, 2017 and July 31, 2020, 2,011,012 patients met our inclusion criteria (pediatric patients [n = 944,559] and adult patients [n = 1,066,453]) (Fig. S1). The mean (SD) ages of the pediatric and adult cohorts were 5.7 (4.8) and 45.4 (18.2), respectively (Table 1). Allergic rhinitis and conjunctivitis were the most common comorbidities in both the pediatric and adult patients. During the observation period, 1.5% of pediatric patients and 4.2% of adult patients received at least one moderate-to-severe AD medication. The median (IQR) duration of observation period was 29.7 (15.6) months and 27.9 (16.0) months for pediatrics and adults, respectively.

**Table 1:** Demographics and clinical characteristics of pediatric (< 18 years) and adult patients (≥ 18 years) with atopic dermatitis.

Characteristics	Pediatrics (n = 944,559)	Adults (n = 1,066,453)
Age (years), mean (SD); median	5.7 (4.8); 4.0	45.4 (18.2); 44.0
Sex, male, n (%)	483,637 (51.2)	452,175 (42.4)
Medical aid recipients, n (%)	17,101 (1.8)	38,081 (3.6)
Region of residence, n (%)		
Metropolitan	173,655 (18.4)	264,668 (24.8)
Urban	294,921 (31.2)	296,705 (27.8)
Rural	475,983 (50.4)	505,080 (47.4)
Comorbidities, n (%)		
Allergic conditions		
Allergic urticaria	162,360 (17.2)	160,852 (15.1)
Allergic rhinitis	624,517 (66.1)	464,593 (43.6)
Asthma	174,446 (18.5)	66,539 (6.2)
Chronic sinusitis	99,133 (10.5)	66,343 (6.2)
Conjunctivitis	260,647 (27.6)	202,112 (19.0)
Skin infections		
Bacterial infections	184,046 (19.5)	181,240 (16.7)
Fungal infections	32,805 (3.5)	125,705 (11.2)
Viral infections	181,952 (19.3)	76,459 (7.2)
Impetigo	78,899 (8.4)	16,836 (1.6)
Eczema Herpeticum	32,487 (3.4)	2308 (0.2)
Skin cancer	–	7979 (0.1)
Neuropsychiatric disorders		
Anxiety	4109 (0.4)	53,588 (5.0)
Depression	2555 (0.3)	49,317 (4.6)
Sleep disorder	1775 (0.2)	54,687 (5.1)
ADHD	5053 (0.2)	998 (0.1)
Cardiovascular comorbidities		
Hypertension	–	189,500 (17.8)
Dyslipidemia	–	162,068 (15.2)
Diabetes	–	110,533 (9.4)
Myocardial infarction	–	3175 (0.3)
Stroke	–	17,441 (1.6)
CCI, mean (SD)	–	0.48 (1.0)
Duration of hospitalization (days), mean (SD)	1.2 (5.8)	1.9 (10.2)
No. of outpatient visits, mean (SD)	18.8 (18.0)	21.7 (26.8)
Moderate-to-severe AD*, n (%)	14,268 (1.5)	44,298 (4.2)
Duration of observation period^†^ (months), mean (SD)	29.2 (9.2)	28.0 (9.2)
Duration of observation period^†^ (months), median (IQR)	29.7 (15.6)	27.9 (16.0)

*AD* atopic dermatitis, *ADHD* attention deficit hyperactivity disorder, *CCI* Charlson comorbidity index, *IQR* interquartile range, *SD* standard deviation.

*Moderate-to-severe AD was defined as receiving at least one immunosuppressant, alitretinoin, intravenous immunoglobulin, dupilumab, or phototherapy, throughout the observational period.

^†^Duration from the first prescription of any AD medication to the end of the study period (Jul 31, 2020).

### Patterns of treatment initiation

Overall, the most common medications prescribed at the initiation of treatment were topical CSs in both pediatric and adult patients, with a prescription rate ranging from 58–85%. (Table 2). The prevalence of individual AD treatments were generally higher in adults than in pediatric patients at treatment initiation. In particular, prescription of systemic CSs was more prevalent in adults than in pediatrics (*p* < 0.001). Conversely, the frequency of topical CSs was higher among the pediatrics compared with adults (*p* < 0.001). Immunosuppressants were rarely prescribed at the initiation of treatment.

**Table 2:** Treatment initiation pattern for atopic dermatitis.

Treatment category	Pediatrics				Adults			*p*-value^‡^
	0–1 year (n = 286,178)	2–5 years (n = 249,070)	6–11 years (n = 263,880)	12–17 years (n = 145,431)	18–39 years (n = 451,924)	40–59 years (n = 357,229)	≥ 60 years (n = 257,300)	
Medications at treatment initiation, mean (SD)	1.30 (0.53)	1.68 (0.70)	1.81 (0.78)	2.05 (0.85)	2.02 (0.90)	1.88 (0.84)	1.77 (0.81)	< 0.001
Prescription with ≥ 2 distinct medications	76,835 (26.8)	136,383 (54.8)	155,244 (58.8)	99,072 (68.1)	294,143 (65.1)	212,333 (59.4)	138,912 (54.0)	< 0.001
Type of medication at treatment initiation								
Topical treatment								
Corticosteroids*	244,439 (85.4)	184,337 (74.0)	191,972 (72.7)	104,294 (71.7)	289,791 (64.1)	207,966 (58.2)	151,614 (58.9)	< 0.001
Low	216,612 (75.7)	133,292 (53.5)	114,994 (43.6)	42,334 (29.1)	97,552 (21.6)	60,366 (16.9)	40,499 (15.7)	< 0.001
Medium	25,996 (9.1)	41,946 (16.8)	53,685 (20.3)	30,811 (21.2)	76,151 (16.9)	55,475 (15.5)	38,919 (15.1)	0.005
High	5,664 (2.0)	14,782 (5.9)	33,512 (12.7)	42,766 (29.4)	144,545 (32.0)	102,435 (28.7)	78,506 (30.5)	< 0.001
Calcineurin inhibitors	335 (0.1)	9551 (3.8)	16,009 (6.1)	11,491 (7.9)	47,167 (10.4)	33,958 (9.5)	21,037 (8.2)	< 0.001
Systemic treatment								
Antihistamines	109,426 (38.2)	172,424 (69.2)	186,313 (70.6)	110,679 (76.1)	327,085 (72.4)	245,826 (68.8)	168,199 (65.4)	< 0.001
Corticosteroids	13,605 (4.8)	39,947 (16.0)	67,498 (25.6)	65,849 (45.3)	228,114 (50.5)	169,640 (47.5)	105,844 (41.1)	< 0.001
Oral	12,953 (4.5)	38,946 (15.6)	65,717 (24.9)	62,635 (43.1)	209,488 (46.4)	148,793 (41.7)	86,826 (33.7)	< 0.001
Median daily dose (IQR)^†^ (mg)	5.0 (5.3)	6.0 (5.0)	7.5 (5.0)	10.0 (5.0)	10.0 (7.5)	10.0 (7.5)	10.0 (7.5)	
Parenteral	1032 (0.4)	2111 (0.9)	5178 (2.0)	11,958 (8.2)	64,414 (14.3)	65,299 (18.3)	47,059 (18.3)	< 0.001
Immunosuppressants								
Cyclosporine	6 (0.0)	18 (0.0)	118 (0.0)	428 (0.3)	3843 (0.9)	3632 (1.0)	2396 (0.9)	< 0.001
Median daily dose (IQR)^†^ (mg)	50.0 (10.0)	50.0 (50.0)	100.0 (50.0)	100.0 (150.0)	100.0 (125.0)	100.0 (150.0)	100.0 (150.0)	
Methotrexate	0	5 (0.0)	6 (0.0)	5 (0.0)	33 (0.0)	49 (0.0)	38 (0.0)	< 0.001
Oral	0	4 (0.0)	5 (0.0)	4 (0.0)	30 (0.0)	44 (0.0)	36 (0.0)	< 0.001
Median weekly dose (IQR)^†^ (mg)	–	10.0 (2.5)	5.0 (7.5)	11.3 (13.8)	10.0 (5.0)	7.5 (5.0)	5.0 (6.9)	
Parenteral	0	1 (0.0)	1 (0.0)	1 (0.0)	3 (0.0)	5 (0.0)	2 (0.0)	0.084
Azathioprine	0	0	1 (0.0)	56 (0.0)	131 (0.0)	94 (0.0)	81 (0.0)	< 0.001
Mycophenolate mofetil	0	1 (0.0)	0	4 (0.0)	6 (0.0)	13 (0.0)	12 (0.0)	< 0.01
Interferon-γ	0	0	1 (0.0)	1 (0.0)	1 (0.0)	2 (0.0)	0	0.329
Intravenous immunoglobulin	4 (0.0)	6 (0.0)	3 (0.0)	4 (0.0)	9 (0.0)	6 (0.0)	2 (0.0)	0.723
Dupilumab	0	0	0	0	0	0	0	n/a
Montelukast	4598 (1.6)	12,335 (5.0)	12,126 (4.6)	2588 (1.8)	5222 (1.2)	5177 (1.5)	2665 (1.0)	< 0.001
Alitretinoin	0	1 (0.0)	5 (0.0)	27 (0.0)	671 (0.2)	874 (0.2)	479 (0.2)	< 0.001
Phototherapy	245 (0.1)	741 (0.3)	2327 (0.9)	2663 (1.8)	10,274 (2.3)	5018 (1.4)	2452 (1.0)	< 0.001

Values are numbers (percentages) unless stated otherwise.

*SD* standard deviation.

*Topical corticosteroids were categorized into three levels based on the WHO potency-based classification of topical corticosteroids: low (class 6–7), medium (class 3–5) and high (class 1–2).

^†^Median daily dose during the observational period. For methotrexate, the median weekly doses were calculated.

^‡^The *p*-values denote comparison between pediatrics and adults.

### Treatment patterns during the entire observational period

The most frequent medications prescribed during the observation period were topical CSs, followed by antihistamines, systemic CSs, and topical CIs (Table 3). The use of antihistamines use was particularly higher among those with allergic comorbidities (Fig. S2). When categorized by potency, those under 11 years of age were more likely to be prescribed low-potency topical CSs, whereas those aged > 12 years and the adults were more likely to be prescribed high-potency topical CSs. Montelukast was more frequently prescribed to pediatrics than adults (*p* < 0.001). Cyclosporine was the most commonly prescribed immunosuppressant, although overall immunosuppressants were rarely prescribed (< 2%). The median daily dose of cyclosporine ranged from 50.0 mg, for children, to 100.0 mg, for adults.

**Table 3:** Treatment pattern for atopic dermatitis during the entire observation period.

Treatment category	Pediatrics				Adults			*p*-value^‡^
	0–1 years (n = 286,178)	2–5 years (n = 249,070)	6–11 years (n = 263,880)	12–17 years (n = 145,431)	18–39 years (n = 451,924)	40–59 years (n = 357,229)	≥ 60 years (n = 257,300)	
Prescriptions for AD treatment per year, mean (SD)	1.27 (1.75)	1.01 (1.32)	1.02 (1.43)	1.43 (2.39)	1.24 (1.99)	1.12 (1.93)	1.34 (2.67)	< 0.0001
Prescriptions with ≥ 2 distinct medications	152,465 (53.3)	169,708 (68.1)	181,334 (68.7)	110,744 (76.1)	324,822 (71.9)	234,390 (65.6)	158,881 (61.7)	< 0.0001
Type of medication								
Topical treatment								
Corticosteroids*	259,788 (90.8)	201,234 (80.8)	207,980 (78.8)	114,420 (78.7)	318,101 (70.4)	226,692 (63.5)	167,042 (64.9)	< 0.0001
Low	238,375 (83.3)	155,542 (62.5)	135,657 (51.4)	56,103 (38.6)	127,202 (28.2)	72,377 (20.3)	49,735 (19.3)	< 0.0001
Medium	49,942 (17.5)	58,837 (23.6)	70,735 (26.8)	43,018 (29.6)	101,190 (22.4)	66,467 (18.6)	47,612 (18.5)	< 0.0001
High	13,695 (4.8)	24,106 (9.7)	48,218 (18.3)	58,122 (40.0)	178,744 (39.6)	120,261 (33.7)	93,504 (36.3)	< 0.0001
Calcineurin inhibitors	6094 (2.1)	17,936 (7.2)	26,565 (10.1)	21,757 (15.0)	70,162 (15.5)	40,657 (11.4)	24,317 (9.5)	< 0.0001
Systemic treatment								
Antihistamines	169,471 (59.2)	192,421 (77.2)	202,088 (76.6)	118,250 (81.3)	347,241 (76.8)	259,277 (72.6)	180,138 (70.0)	< 0.0001
Corticosteroids	36,509 (12.8)	58,827 (23.6)	87,152 (33.0)	79,244 (54.5)	258,709 (57.2)	186,284 (52.1)	119,100 (46.3)	< 0.0001
Oral	35,104 (12.3)	57,444 (23.1)	84,883 (32.2)	75,744 (52.1)	239,681 (53.0)	164,635 (46.1)	98,660 (38.3)	< 0.0001
Median daily dose (IQR)^†^ (mg)	5.0 (5.3)	5.0 (5.1)	7.5 (5.0)	10.0 (5.0)	10.0 (5.0)	10.0 (10.0)	10.0 (10.0)	
Parenteral	3114 (1.1)	3926 (1.6)	9163 (3.5)	20,165 (13.9)	88,377 (19.6)	78,477 (22.0)	58,092 (22.6)	< 0.0001
Immunosuppressants								
Cyclosporine	38 (0.0)	87 (0.0)	509 (0.2)	1739 (1.2)	8419 (1.9)	5542 (1.6)	3449 (1.3)	< 0.0001
Median daily dose (IQR)^†^ (mg)	50.0 (24.0)	50.0 (40.0)	75.0 (50.0)	100.0 (150.0)	100.0 (125.0)	100.0 (150.0)	100.0 (150.0)	
Methotrexate	1 (0.0)	11 (0.0)	21 (0.0)	57 (0.0)	384 (0.1)	182 (0.1)	119 (0.0)	< 0.0001
Oral	1 (0.0)	10 (0.0)	20 (0.0)	56 (0.0)	373 (0.1)	176 (0.1)	113 (0.0)	< 0.0001
Median weekly dose (IQR)^†^ (mg)	7.5 (-)	10.0 (5.0)	7.5 (5.0)	10.0 (5.0)	10.0 (5.0)	10.0 (5.0)	7.5 (5.0)	
Parenteral	0 (0.0)	5 (0.0)	2 (0.0)	1 (0.0)	11 (0.0)	7 (0.0)	8 (0.0)	0.0062
Azathioprine	0	0	4 (0.0)	83 (0.1)	232 (0.1)	141 (0.0)	116 (0.0)	< 0.0001
Mycophenolate mofetil	0	2 (0.0)	2 (0.0)	5 (0.0)	16 (0.0)	29 (0.0)	25 (0.0)	< 0.0001
Interferon-γ	0	3 (0.0)	3 (0.0)	8 (0.0)	11 (0.0)	2 (0.0)	0	0.6112
Intravenous immunoglobulin	18 (0.0)	10 (0.0)	5 (0.0)	6 (0.0)	10 (0.0)	7 (0.0)	3 (0.0)	0.0032
Dupilumab	0	0	1 (0.0)	24 (0.0)	147 (0.0)	31 (0.0)	4 (0.0)	< 0.0001
Montelukast	18,747 (6.6)	23,165 (9.3)	19,243 (7.3)	4359 (3.0)	8144 (1.8)	7709 (2.2)	4113 (1.6)	< 0.0001
Alitretinoin	0	1 (0.0)	13 (0.0)	84 (0.1)	1149 (0.3)	1286 (0.4)	658 (0.3)	< 0.0001
Phototherapy	954 (0.3)	1761 (0.7)	4331 (1.6)	5109 (3.5)	15,925 (3.5)	6529 (1.8)	3410 (1.3)	< 0.0001

Values are numbers (percentages) unless stated otherwise.

*AD* atopic dermatitis, *SD* standard deviation, *IQR* interquartile range.

*Topical corticosteroids were categorized into three levels based on the WHO potency-based classification of topical corticosteroids: low (class 6–7), medium (class 3–5) and high (class 1–2).

^†^Median daily dose during the observational period. For methotrexate, the median weekly doses were calculated.

^‡^The *p*-values denote comparison between pediatrics and adults.

For characteristics associated with oral CSs use, adults had higher odds compared with pediatrics (adjusted OR 2.84, 95% CI 2.82–2.86) and patients with allergic rhinitis were more likely to be prescribed oral CSs (1.18, 1.17–1.19) (Table S2).

### Discontinuation of AD treatment

**Figure 1:**
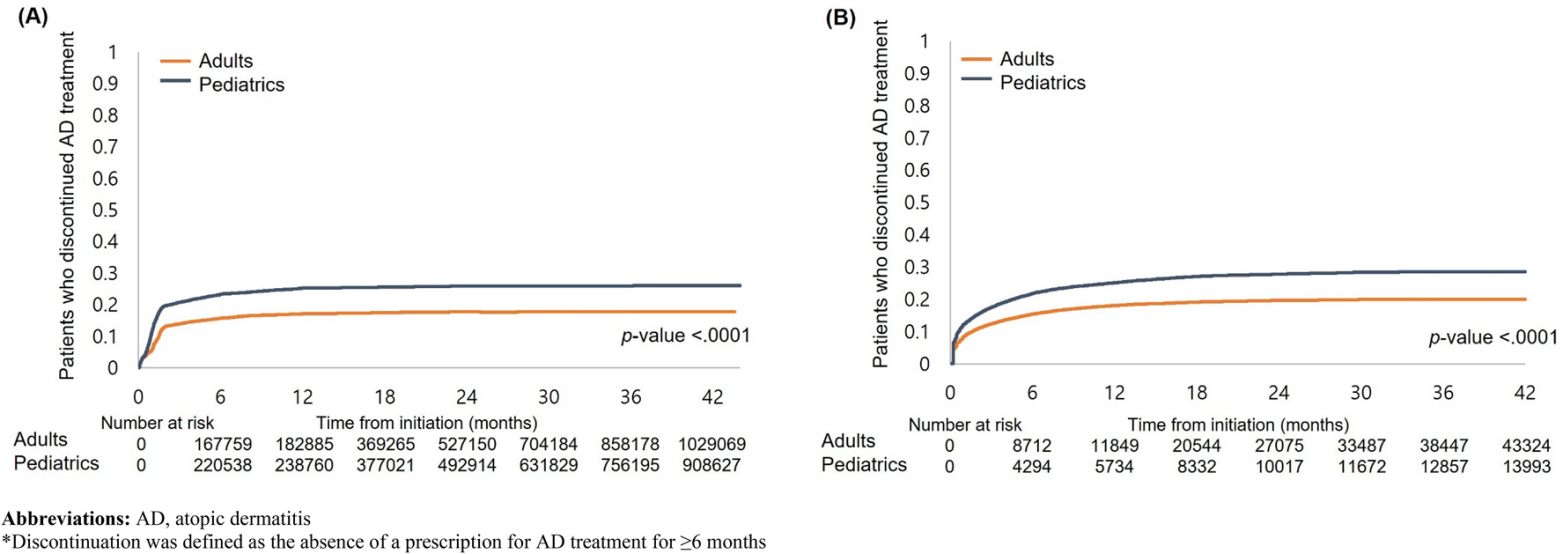
Kaplan–Meier curve for time to discontinue* (**A**) AD treatment among pediatrics and adult cohort and (**B**) moderate-to-severe AD treatment among those who were prescribed moderate-to-severe AD treatment. *AD* atopic dermatitis. *Discontinuation was defined as the absence of a prescription for AD treatment for ≥ 6 months.

Figure 1A,B shows the Kaplan–Meier curves for time to discontinuation in the pediatric and adult cohorts. The overall probability of AD treatment discontinuation was significantly higher in pediatric patients than in adults (*p* < 0.001) (Fig. 1A). Specifically, the estimated rate of treatment discontinuation was 23.3% during the first 12 months for pediatric patients and 15.8% for adults. Furthermore, the probability of moderate-to-severe AD treatment discontinuation was higher in the pediatric group than in the adult group (*p* < 0.001) (Fig. 1B). The estimated rate of discontinuing moderate-to-severe AD treatment within the first 12 months was 21.8% in pediatric patients and 15.4% in adults.

For characteristics associated with treatment discontinuation, adults were less likely to discontinue AD treatment compared with pediatrics (adjusted OR 0.57, 95% CI 0.56–0.58) and patients with allergic rhinitis had lower odds of treatment discontinuation (0.85, 0.84–0.86) (Table S2).

### Restart after discontinuation

**Figure 2:**
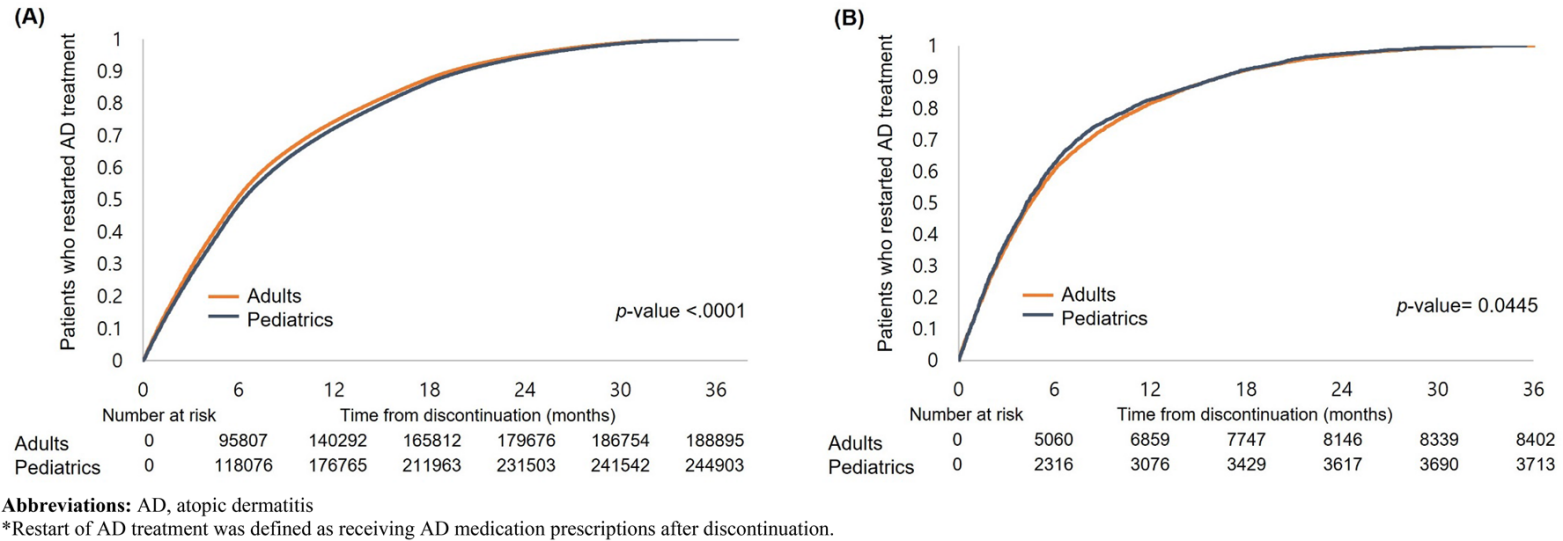
Kaplan–Meier curve for time to restart* after discontinuation of (**A**) AD treatment among pediatrics and adult cohort and (**B**) moderate-to-severe AD treatment among those who were prescribed moderate-to-severe AD treatment. *AD* atopic dermatitis. *Restart of AD treatment was defined as receiving AD medication prescriptions after discontinuation.

Figure 2A,B presents the Kaplan–Meier curves for the probability of restarting AD treatment among the discontinuers in the pediatric and adult cohorts. There was a significant difference in the probability of restarting any AD treatment among the discontinuers in pediatrics and adults, with a median time to restart of 368 and 356 days, respectively (Fig. 2A). Additionally, restarting tended to be earlier in patients taking medications for moderate-to-severe AD (Fig. 2B). The median time to restart of treatment for moderate-to-severe AD among those who discontinued moderate-to-severe AD treatment was 308 and 316 days for pediatric and adult patients, respectively.

## Discussion

This study aimed to assess real-world treatment patterns of patients with AD using a nationwide claims database in South Korea. The results showed significant differences in the treatment pattern between the pediatrics and adults patients with AD. Overall, topical CSs were the most commonly prescribed medications, both at the initiation of treatment and during the entire observation period in all age groups. This is in line with the results of previous studies that analyzed treatment patterns among children and adults with AD in the United States^11^. Not surprisingly, relevant guidelines also suggest topical CSs as the first-line treatment and maintenance therapy^12,13^. Of note, infants (0–1 years) and young children (2–5 years) had a higher prescription for topical CSs than other age groups despite the widespread steroid phobia among the parents of pediatric patients with AD^14^. Furthermore, we assessed the prevalence of topical CSs based on potency. We found that patients aged < 11 years were more likely to be prescribed low-potency topical CSs, whereas those aged > 12 years and the adults were more likely to be prescribed high-potency topical CSs. This may be attributed to the fact that physicians consider age before prescription; additionally, it can be assumed that the AD lesions occurring in infants and children are not as severe as those in adults.

Antihistamines were the second most commonly prescribed medications in all age groups, with a prescription rate ranging from 59 to 81%. In a study of the US medical claims database, antihistamines were prescribed to 40–50% of pediatric patients with AD^7^. Another study conducted in a tertiary hospital in India reported that 43% of patients with AD were prescribed antihistamines^15^. Although little to no evidence exists regarding the role of antihistamines in AD treatment, they are widely prescribed by physicians, perhaps, to reduce the itchiness that AD causes. However, it should be noted that the antipruritic effect of antihistamines cannot adequately alleviate AD, and additional or other treatments should be considered^16^. In addition, the current guidelines only recommend that antihistamines should be used for the purpose of sedation if a patient has sleep disturbance or other atopic comorbidities^5^. Another possible explanation for the high use of antihistamines would be the high proportion of patients with allergic comorbidities among those who were prescribed antihistamines, and this suggests that physicians may have prescribed them to concomitantly treat the pruritus of AD and other allergic comorbidities. Similarly, regular use of montelukast, specifically among the pediatric patients may be explained by the high prevalence of allergic rhinitis in patients with AD, along with the absence of major adverse effects of montelukast in children^17^.

Systemic CSs are also frequently prescribed to patients with AD, even at treatment initiation, although most guidelines discourage their use as a first-line therapy^18^. This finding is similar to the results of previous studies that reported a high prevalence of systemic CS use^19,20^. Taken together, systemic CSs are still widely prescribed for the treatment of AD despite unfavorable risk–benefit profiles, which indicates the need for safer and more effective systemic treatments^21^.

In this study, immunosuppressants were not commonly prescribed to either pediatric or adult patients. Among the medications, cyclosporine was the most frequently prescribed immunosuppressant, particularly in adults. This is in line with the current guidelines that recommend cyclosporine as the first-line therapy to treat severe refractory AD and the survey study that found cyclosporine as the preferred first-line therapy among the European dermatologists^22,23^. However, a previous study conducted in the UK reported methotrexate and azathioprine as the most frequently prescribed immunosuppressants, although their use is off-label^24^. Another study based on US pediatric and German populations also showed that methotrexate is prescribed more frequently than cyclosporine^25^. Discrepancies in the findings of the previous studies and those of the present study may be explained by the heterogeneity in the study populations and the medical environment. The prevalence of liver disease is relatively high among South Koreans, and hence, physicians might have been reluctant to prescribe methotrexate, which has a hepatotoxic risk profile^26^. In addition, previous studies have shown that Asian patients with AD present a unique phenotype involving increased hyperplasia and the increased activation of Th17 and Th2/Th22^8,27^. Differences in phenotypes and serum biomarkers between Asian and European American patients with AD may affect the treatment response rate, which, in turn, influences the decision of physicians in prescribing medications. Moreover, in countries with different access to healthcare systems, cyclosporine, which requires regular monitoring of blood pressure and renal function, may not be commonly prescribed^28^.

The most notable change in guidelines for the treatment of AD in South Korea is the recommendation of dupilumab as an important treatment option for patients with moderate-to-severe AD^29,30^. Likewise, a recent study based on German AD registry reported dupilumab to be the leading treatment among the patients with moderate-to-severe AD^31^. However, the use of dupilumab was minimal in the current study, perhaps due to the reimbursement date of dupilumab in South Korea. Thus, future studies based on the latest data are in need to evaluate the prescription pattern of dupilumab.

In the present study, approximately 25% of pediatric patients and 15% of adults discontinued AD treatment during the observation period. In both the groups, the majority of patients who discontinued AD treatment did so within the first 12 months of treatment. Information on the reasons for discontinuation were not included in the database; however, one possible reason is that due to the chronic and episodic nature of AD, patients may discontinue their treatment during the remission period and restart at subsequent flare-ups^32^. The median time to restart of AD treatment since discontinuation was approximately 1 year for both adults and pediatrics, although adults tended to restart slightly earlier than pediatrics. Restarting the treatment for moderate-to-severe AD appeared to be earlier than that for overall AD, which suggests that moderate-to-severe AD patients have a shorter time to recurrence.

This study had several limitations. First, owing to the nature of the claims data, we identified patients with AD using the diagnostic code; it was not possible to use the Hanifin and Rajka criteria, which are the diagnostic standard for AD. The HIRA database does not include data on symptoms or clinical manifestations; hence, disease severity was defined based on the prescribed medications, not the Eczema Area and Severity Index. Second, investigating the reasons for discontinuation or restart was beyond the scope of our study because of the lack of information about the reasons in our data. Further studies are necessary to determine why patients discontinue (e.g., adverse events and ineffectiveness) or restart AD treatment. Finally, we cannot rule out the possibility that the medications were prescribed to treat conditions other than AD. To address this concern, we included only prescriptions with a primary diagnosis code for AD in our analysis.

Despite these limitations, this study is significant in that it analyzed the real-world treatment patterns for AD in over 2 million patients using the nationwide data that covers the entire South Korean population. Additionally, we used all AD prescription records in Korea, which allowed us to estimate prevalence according to the severity of AD based on medication use. This study will improve the understanding of the latest treatment patterns for AD in clinical practice, particularly among the Asian population wherein research is sparse.

## Supplementary Information

Supplementary Information.

## Data Availability

The datasets generated during and/or analysed during the current study are not publicly available due to Korean Health Insurance Review and Assessment Service policy but are available from the corresponding author on reasonable request.
