# Supplementary material for: Real-world treatment patterns for atopic dermatitis in South Korea
Source: Sci Rep. 2022 Aug 10;12:13626. doi: 10.1038/s41598-022-17222-y (PMC9365828; doi:10.1038/s41598-022-17222-y)
Supplement: Supplementary file 1 — Supplementary Information. [file 41598_2022_17222_MOESM1_ESM.docx]

**Real-World Treatment Patterns for Atopic Dermatitis in South Korea**

Jihyun Lee MD, PhD^1†^, Ahhyung Choi PharmD^2†^, Yunha Noh PharmD, PhD^2,3^, In-Sun Oh PhD^2,3^, Ja-Young Jeon PhD^4^, Hyun-Jeong Yoo MD^4^, Ju-Young Shin PhD^2,3,5*^, Sang Wook Son MD, PhD^6*^

^†^These authors contributed equally to this work.

^*^These authors are co-corresponding authors of this work.

^1^ Department of Dermatology, Seoul St. Mary’s Hospital, College of Medicine, The Catholic University of Korea, Seoul, South Korea

^2^ School of Pharmacy, Sungkyunkwan University, Suwon, South Korea

^3^ Department of Biohealth Regulatory Science, Sungkyunkwan University, Suwon, South Korea

^4^ Pfizer Pharmaceuticals Korea Ltd., Seoul, South Korea

^5^ Department of Clinical Research Design & Evaluation, Samsung Advanced Institute for Health Sciences & Technology, Sungkyunkwan University, Seoul, South Korea

^6^ Department of Dermatology, Korea University College of Medicine, Seoul, South Korea.

**Correspondence to:** Ju-Young Shin, PhD, Associate Professor

School of Pharmacy, Sungkyunkwan University,

2066 Seobu-ro, Jangan-gu, Suwon, Gyeonggi-do 16419, South Korea

Tel: 82-31-290-7702; Fax: 82-31-292-8800; E-mail: [shin.jy@skku.edu](mailto:shin.jy@skku.edu)

**Correspondence to:** Sang Wook Son, MD, PhD, Professor

Department of Dermatology, Korea University College of Medicine,

46 Gaeunsa 2-gil, Seongbuk-gu, Seoul 02842, South Korea.

Tel: 82-31-412-5180, Fax: 82-31-412-4208; E-mail: [skin4u@korea.ac.kr](mailto:skin4u@korea.ac.kr)

**Table S1.** List of immune-mediated inflammatory diseases

| **Immune-mediated inflammatory diseases** |
| --- |
| Inflammatory bowel disease |
| Lupus erythematosus |
| Rheumatoid arthritis |
| Juvenile idiopathic arthritis |
| Psoriasis |
| Ankylosing spondylitis |
| Polymyositis |
| Sjogren’s syndrome |
| Systemic sclerosis |
| Thromboangiitis Obliterans |
| Behcet's disease |
| Sarcoidosis |
| Pemphigus |
| Vitiligo |

**Figure S1.** Study flow chart


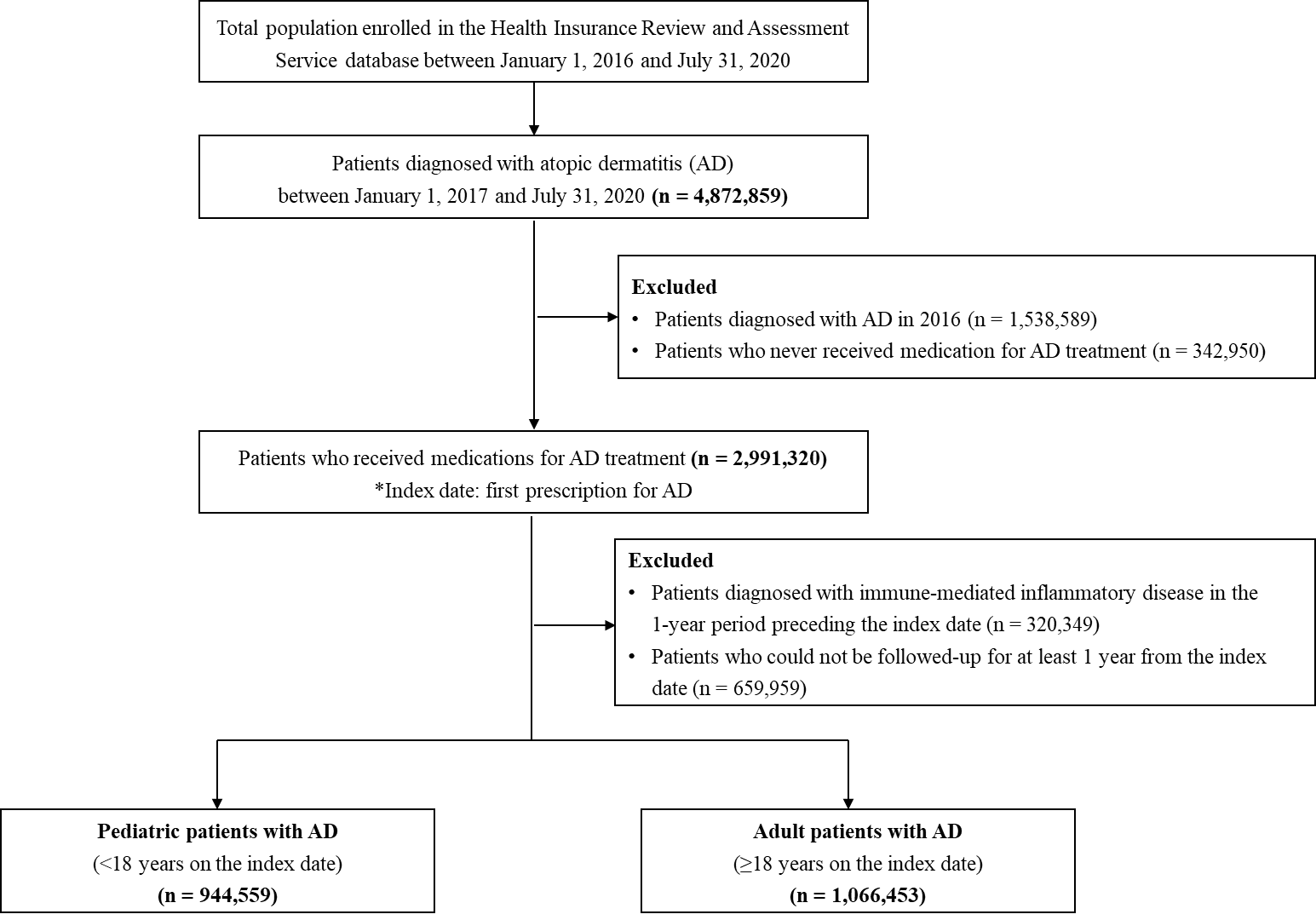


**Abbreviations:** AD, atopic dermatitis;

**Figure S2.** Prevalence of antihistamines use among the patients with AD, stratified by allergic comorbidities


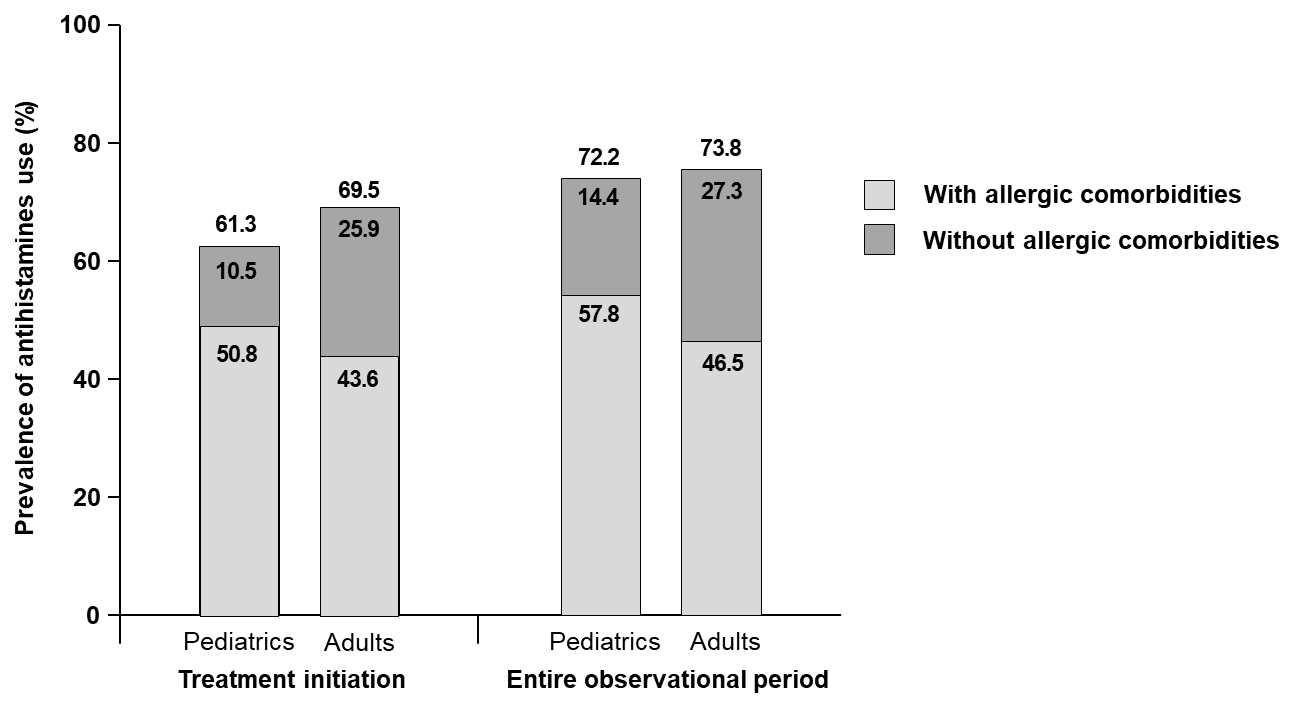


**Abbreviations:** AD, atopic dermatitis

**Table S2.** Adjusted ORs (95% CI) for oral CSs use and treatment discontinuation in patients with AD

|  | Oral CSs use,  adjusted OR* (95% CI) | Treatment discontinuation,  adjusted OR* (95% CI) |
| --- | --- | --- |
| **Age** |  |  |
| < 18 years | 1.0 (ref) | 1.0 (ref) |
| ≥ 18 years | 2.84 (2.82-2.86) | 0.57 (0.56-0.58) |
| **Sex** |  |  |
| Male | 1.0 (ref) | 1.0 (ref) |
| Female | 0.95 (0.94-0.96) | 1.00 (0.99-1.01) |
| **Comorbidities (Yes vs. no)** |  |  |
| Allergic urticaria | 1.07 (1.06-1.09) | 1.07 (1.06-1.09) |
| Allergic rhinitis | 1.18 (1.17-1.19) | 0.85 (0.84-0.86) |
| Asthma | 0.84 (0.83-0.85) | 1.05 (1.04-1.06) |
| Chronic sinusitis | 1.01 (0.99-1.02) | 0.97 (0.95-0.99) |
| Conjunctivitis | 0.95 (0.94-0.96) | 1.04 (1.02-1.05) |

**Abbreviations:** CI, confidence interval; CS, corticosteroid; AD, atopic dermatitis
* Adjusted for age, sex and allergic comorbidities
